# Identification of pannexin 1-regulated genes, interactome, and pathways in rhabdomyosarcoma and its tumor inhibitory interaction with AHNAK

**DOI:** 10.1038/s41388-020-01623-2

**Published:** 2021-02-09

**Authors:** Xiao Xiang, Stéphanie Langlois, Marie-Eve St-Pierre, Anna Blinder, Philippe Charron, Tyson E. Graber, Stephanie L. Fowler, Stephen D. Baird, Steffany A. L. Bennett, Tommy Alain, Kyle N. Cowan

**Affiliations:** 1 Molecular Biomedicine Program, Children’s Hospital of Eastern Ontario Research Institute, Ottawa, ON Canada; 2 Department of Cellular and Molecular Medicine, University of Ottawa, Ottawa, ON Canada; 3 Department of Surgery, Children’s Hospital of Eastern Ontario, University of Ottawa, Ottawa, ON Canada; 4 Department of Biochemistry, Microbiology and Immunology, University of Ottawa, Ottawa, ON Canada; 5 Neural Regeneration Laboratory and Ottawa Institute of Systems Biology, University of Ottawa, Ottawa, ON Canada; 6 UK Dementia Research Institute, University College London, London, UK

**Keywords:** Proteomics, Paediatric cancer, Sarcoma

## Abstract

Rhabdomyosarcoma (RMS), the most common soft tissue sarcoma in children, is an aggressive cancer with a poor prognosis. Despite current management, the 5-year survival rate for patients with metastatic RMS is ∼30%; underscoring the need to develop better treatment strategies. We have recently reported that pannexin 1 (PANX1) levels are downregulated in RMS and that restoring its expression inhibits RMS progression. Here, we have surveyed and characterized the molecular changes induced by PANX1 re-expression in RMS. We cataloged transcriptomic changes in this context by RNA sequencing. At the protein level, we unveiled PANX1 interactors using BioID, complemented by co-immunoprecipitation coupled to high-performance liquid chromatography/electrospray ionization tandem mass spectrometry performed in PANX1-enriched fractions. Using these data, we generated searchable public databases for the PANX1 interactome and changes to the RMS transcriptome occurring when PANX1 expression is restored. STRING network analyses revealed a PANX1 interactome involving plasma membrane and cytoskeleton-associated proteins including the previously undescribed interactor AHNAK. Indeed, AHNAK knockdown abrogated the PANX1-mediated reduction in RMS cell viability and migration. Using these unbiased approaches, we bring insight to the mechanisms by which PANX1 inhibits RMS progression, identifying the cell migration protein AHNAK as a key modifier of PANX1-mediated changes in RMS malignant properties.

## Introduction

Rhabdomyosarcoma (RMS) is the most common soft tissue sarcoma in childhood [1]. RMS tumors are typically associated with the skeletal muscle lineage, displaying two major subtypes: embryonal (eRMS) and alveolar (aRMS) [2]. While eRMS is generally associated with a favorable clinical outcome, aRMS is more aggressive and often metastatic [3]. The 5-year survival of patients with metastatic RMS is below 30% and despite the use of invasive multimodal treatment regimens, their prognosis has not improved in the last 30 years [1, 4]. The discovery of novel therapeutic strategies for RMS is thus of utmost importance.

RMS is thought to originate as a consequence of regulatory disruption of myogenic precursor cell differentiation [5, 6]. We recently described a key role for pannexin 1 (PANX1) in skeletal myogenesis [6, 7]. Pannexins (PANX1, PANX2, and PANX3) are single membrane channels that allow the release of small molecules such as nucleotides [8]. Our work has shown that PANX1 levels are low in undifferentiated skeletal muscle myoblasts and increase during myogenesis in vitro and in vivo [6, 9]. Blocking PANX1 channel activity inhibited myoblast differentiation, while PANX1 overexpression promoted this process [6]. Based on this, we explored whether PANX1 levels are altered in RMS and if so, whether restoration of its expression could reduce RMS malignant properties. We found that PANX1 expression is downregulated in patient-derived RMS cell lines and tumor specimens when compared to differentiated skeletal muscle cells and tissue [10]. Increasing PANX1 levels abrogated the proliferative and migratory potential of eRMS and aRMS cells, inhibited 3D tumor spheroid growth and induced regression of established spheroids through the induction of apoptosis [10]. Moreover, while control tumors grew rapidly in mice, PANX1 overexpression significantly reduced eRMS and aRMS growth [10]. As ectopic PANX1 effectively alleviates RMS tumor growth, deciphering its downstream signaling pathways would bring insight into the molecular mechanism by which PANX1 reduces RMS malignant properties while offering an opportunity to identify potential new therapeutic targets.

PANX1 is a type III multi-pass transmembrane glycoprotein with both the amino- and carboxyl-termini inside the cell [11]. PANX1 channels have been implicated in various pathological conditions including ischemia [12], stroke [13], diabetes [14], epilepsy [15], and hypertension [16] as well as cancers such as glioma [17] and melanoma [18, 19]. While the underlying signaling mechanism of PANX1 under pathological conditions remains largely unknown, it has been primarily attributed to disrupted purinergic and/or adrenergic signaling via ATP or other nucleotides [20]. However, PANX1 has been recently implicated in the Wnt/β-catenin signaling pathway in melanoma [19]. In addition, modulation of PANX1 expression has been shown to alter the expression of E-cadherin, vimentin and matrix metalloproteinase-9 via the extracellular signal-regulated kinase 1/2 signaling pathway in testicular cancer cells [21]. Although ATP release has been central in PANX1 research [20], it has become increasingly evident that the interaction of PANX1 with various signaling molecules mediates key cellular processes. For instance, the interaction of PANX1 with actin-related proteins 2/3 has been proposed to regulate the actin-mediated mechanical force generation [22], and the sequestration of collapsin response mediator protein-2 by Panx1 channels regulates microtubule remodeling [23]. While the molecular mechanisms by which PANX1 functions have started to be unveiled in some contexts, the signaling pathways involved in PANX1-mediated inhibition of RMS progression had yet to be investigated.

In the present study, we show that PANX1 overexpression in RMS cells invoked transcriptomic changes in a number of biological processes (BPs) such as apoptosis and cellular migration, as well as in the MAPK and Rap1 signaling pathways. Using BioID, we revealed the PANX1 interactome in Rh18 (eRMS) and Rh30 (aRMS) patient-derived cell lines. Common PANX1 interactors between the two RMS subtypes were associated with the plasma membrane and cytoskeleton. The BioID PANX1 interactome was further compared to and corroborated with a complementary set of PANX1 binding partners identified by HPLC-ESI-MS/MS analysis from PANX1-enriched subcellular fractions. We validated the novel interaction of PANX1 with AHNAK, which was identified here as the top PANX1 interactor. AHNAK is a large scaffolding protein that has been linked to migration and invasion in other cancers [24–27]. Knockdown of AHNAK in PANX1-expressing Rh18 and Rh30 cells abrogated the PANX1-mediated reduction in cell viability, migration, and increase in anoikis, suggesting that PANX1 regulation of RMS tumor malignant properties involves its interaction with AHNAK. Using data generated through these genome-wide unbiased approaches, we have also generated the first PANX1 transcriptomic and proteomic public searchable databases for easy access to our entire RNA-seq and BioID data, which may foster new research avenues identifying pathways regulating PANX1 and its functions, as well as potential clinical translation toward novel therapeutic strategies.

## Results

### Identification of the PANX1 transcriptome in RMS using RNA-Seq

**Fig. 1:**
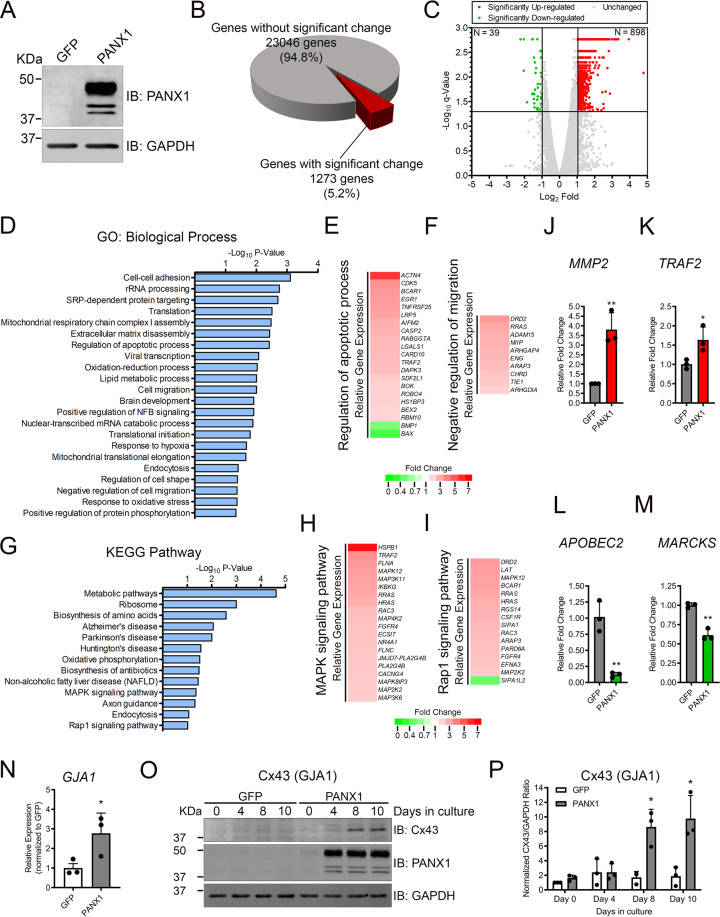
RNA-seq analysis of PANX1-expressing aRMS cells. RNA was extracted from stable Rh30 cells expressing PANX1 or the GFP control for RNA-seq analysis 48 h post cumate induction. **A** Representative western blots of stable Rh30 cells expressing PANX1 and its GFP control prior to RNA extraction. Western blots are representative of three independent experiments. **B** Pie chart highlighting the number of genes identified by RNA-seq (*n* = 3). Genes with an FDR cut-off of *q* < 0.05 after differential expression test are considered significantly changed. **C** Volcano plot showing gene expression profiles. Up- (Log_2_ Fold > 1) and downregulated (Log_2_ Fold < −1) genes are shown in red and green, respectively. The horizontal bar indicates the FDR cut-off (*q* = 0.05). **D** DAVID gene ontology (GO) analysis of the significantly up- and downregulated genes. A list of enriched GO terms in biological processes is shown. The gene hits under GO: Regulation of apoptotic process (**E**) and GO: Negative regulation of migration (**F**) are shown along with their expression in cells expressing PANX1 relative to their respective GFP controls. **G** KEGG pathway analysis of the significantly up- and downregulated genes, of which the relative expression of the genes implicated in MAPK signaling (**H**) and Rap1 signaling (**I**) pathways are shown. RT-qPCR validation of RNA-seq hits *MMP2* (**J**), *TRAF2* (**K**), *APOBEC2* (**L**), and *MARCKS* (**M**). Red and green bars indicate up- or downregulation from RNA-seq. Results are expressed as mean ± s.d. of three independent experiments. *P < 0.05 and **P < 0.01 compared to GFP. *GJA1*, which encodes Cx43, showing significantly increased transcript levels (**N**) from RNA-seq, and validation of its temporal increase in protein levels by western blotting (**O**) and its quantification (**P**) in Rh30 cells expressing PANX1 compared to the GFP control. *P < 0.05 compared to GFP on Day 8 and Day 10 in (**O**). Results are expressed as mean ± s.d. of three independent experiments.

We have previously shown that PANX1 overexpression in RMS cells reduced their proliferation, migration, and inhibited tumor growth via induction of apoptosis [10]. As our first step toward understanding the PANX1 downstream signaling in RMS, Rh30 (aRMS) cells expressing ectopic PANX1 or control GFP (Fig. 1A) were subjected to RNA-seq. The subsequent differential expression (DE) analysis identified 1273 genes (5.2%) significantly changed in PANX1 overexpressing cells with a false discovery rate less than 0.05 (*q* < 0.05) (Fig. 1B). Amongst the 1273 significantly changed genes, 39 were downregulated (Log_2_ Fold expression change < −1) and 898 were upregulated (Log_2_ Fold expression change > 1), which are shown in the upper left and right quadrants of the volcano plot, respectively (Fig. 1C). The significantly regulated genes were classified by gene ontology (GO) according to BP using DAVID [28] (Fig. 1D), which revealed numerous highly enriched (Fisher’s Exact *P* value < 0.05) GO_BP terms in accordance with our previous observations [10], including regulation of apoptotic processes (Fig. 1E) and negative regulation of migration (Fig. 1F). In addition, KEGG pathway analysis results (Fig. 1G) indicated that MAPK (Fig. 1H) and Rap1 (Fig. 1I) were amongst the most enriched (Fisher’s exact *P* value < 0.05) signaling pathways in PANX1-expressing cells. To validate the RNA-seq results, we selected two upregulated gene hits, matrix metalloprotease 2 (*MMP2*) and TNF receptor-associated factor 2 (*TRAF2*), and two downregulated gene hits, apolipoprotein B mRNA-editing enzyme catalytic subunit 2 (*APOBEC2*) and myristoylated alanine rich C-kinase substrate (*MARCKS*), for RT-qPCR validation. Our results showed that *MMP2* (Fig. 1J) and *TRAF2* (Fig. 1K) were significantly upregulated, while *APOBEC2* (Fig. 1L) and *MARCKS* (Fig. 1M) were significantly downregulated when PANX1 was expressed, which were all concordant with the RNA-seq results. The upregulated *GJA1* gene (Fig. 1N) was further investigated as ectopic expression of its protein product connexin 43 (Cx43) in RMS cells was previously shown to elicit a tumor-suppressive phenotype [29] similar to that of PANX1 [10]. Western blot analysis (Fig. 1O, P) showed a significant increase in Cx43 levels (unphosphorylated/poorly phosphorylated species (*P*_0_)) in PANX1-expressing Rh30 cells compared to control cells while the 20 kDa variant of Cx43 (Cx43-20K) was not detected under these conditions (data not shown). Using this unbiased genome-wide approach, our transcriptomic analysis identified the genes that are regulated in PANX1-expressing RMS cells together with the key cellular processes in which they may be involved.

### The BirA* tag does not interfere with PANX1 function

**Fig. 2:**
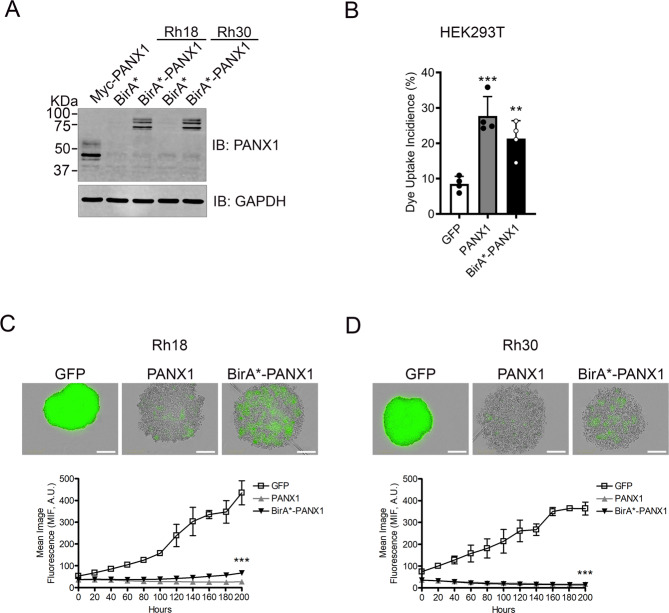
The BirA* tag does not affect PANX1 function. **A** Representative western blot of BirA*-PANX1 and Myc-PANX1 expressed in Rh18 (eRMS) and Rh30 (aRMS) cells. Western blots are representative of three independent experiments. **B** Sulforhodamine B dye uptake induced by mechanical stimulation in HEK293T cells expressing GFP (control), PANX1, or BirA*-PANX1. Results are expressed as mean ± s.d. of four independent experiments. ***P < 0.001 and **P < 0.01 compared to GFP. 3D spheroid formation using our stable cumate-inducible Rh18 and Rh30 cell lines was assessed in the presence of cumate to allow PANX1 or BirA*-PANX1 expression. Representative images of Rh18 (**C**) and Rh30 (**D**) cells taken at 200 h are shown. As these cells express GFP constitutively, the changes in mean image fluorescence (MIF) over time, a measurement of spheroid size, were quantified for both Rh18 (**C**) and Rh30 (**D**) cells. Results are expressed as mean ± s.d. of three independent experiments. ***P < 0.001 compared to GFP for both PANX1 and BirA*-PANX1. A.U.: arbitrary units. Bars = 300 µm.

As many BPs are mediated through protein–protein interactions [30], and as transcriptomic data may not fully recapitulate the proteome of a cell, we next used BioID [31] to unveil PANX1-interacting proteins and gain further insight into the molecular mechanism by which PANX1 functions in RMS. The carboxyl terminus of PANX1 was fused with a promiscuous biotin ligase found in *Escherichia coli*, BirA*, and expressed in RMS cells. With the addition of exogenous biotin, BirA* catalyzes the biotinylation of the interacting partners in a distance-dependent manner. The biotin labeled proteins can be denatured and captured by streptavidin-mediated pull-down and subsequently identified by mass spectrometry. This method offers advantages such as capturing proteins with weak or transient interactions that could be missed by co-immunoprecipitation [31]. BirA*-PANX1 showed a banding pattern similar to that of Myc-PANX1 by western blotting (Fig. 2A). PANX1 is detected as multiple bands due to post-translational modifications [32]. As expected, Myc-PANX1 was detected as three main species with apparent molecular weights of ~43, 45, and 53 kDa, while BirA*-PANX1 was detected as ~69, 72, and 81 kDa bands, showing an average increase of ~4 kDa (Myc predicted: 3.3 kDa [10]) and ~31 kDa (BirA* predicted: 35 kDa [31]), respectively, relative to untagged wild-type PANX1 (detected at ~39, 40, and 49 kDa in these cells) [10]. To ensure that BirA* does not affect PANX1 function, we first performed a sulforhodamine B dye uptake assay in HEK293T cells, devoid of endogenous pannexins, transiently expressing PANX1, BirA*-PANX1, or the GFP control vector. PANX1 and BirA*-PANX1-induced similar levels of dye uptake, which were both significantly higher than that of cells expressing the control vector (Fig. 2B). We then generated stable Rh18 (eRMS) and Rh30 (aRMS) cell lines using our previously described cumate-inducible system [10] to express BirA*-PANX1 and performed a 3D spheroid formation assay. As we previously reported, all cells express GFP constitutively under this expression system but express PANX1 or BirA*-PANX1 only in the presence of cumate [10]. In this spheroid assay, cumate was added at the time of cell seeding. Aggregation and compaction in GFP control, PANX1-, and BirA*-PANX1-expressing Rh18 (Fig. 2C) and Rh30 (Fig. 2D) cells were observed 48 h after seeding. Using the constitutively expressed GFP to quantify spheroid size in all conditions, we show that the expression of both PANX1 and BirA*-PANX1 prevented Rh18 (Fig. 2C) and Rh30 (Fig. 2D) 3D spheroid growth compared to their respective GFP control cells. Indeed, PANX1- and BirA*-PANX1-expressing cells gradually lost their constitutive GFP fluorescence likely due to cell death [10]. All together showing that the BirA* tag does not interfere with PANX1 functions.

### BioID analysis identified plasma membrane and cytoskeletal PANX1-interacting proteins in RMS cells

**Fig. 3:**
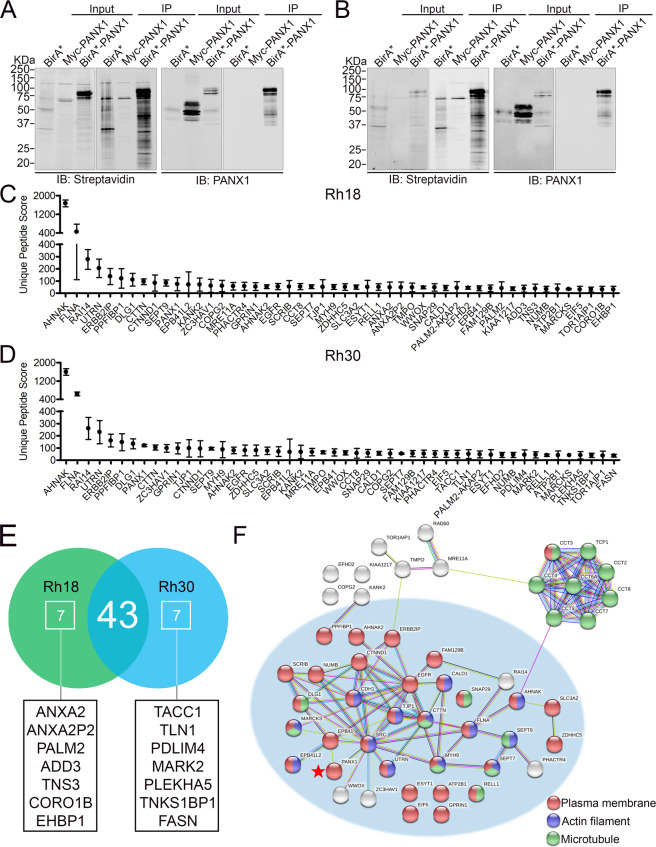
BioID analysis of PANX1-interacting proteins in RMS cells. Representative western blots of biotinylated proteins captured by streptavidin and BirA*-PANX1 in Rh18 (eRMS) (**A**) and Rh30 (aRMS) (**B**) are shown. BirA* and Myc-PANX1 were used as background controls for biotinylation and HPLC-ESI-MS/MS, respectively. Western blots are representative of three independent experiments. Top 50 protein hits in Rh18 (**C**) and Rh30 (**D**) identified by BioID are displayed. Results are shown as mean ± s.d. of Unique Peptide Counts normalized to the Myc-PANX1 background controls from three independent experiments. **E** Venn diagram showing the overlapping top 50 hits between Rh18 and Rh30 cells. **F** STRING analysis of the 43 overlapping hits reveals a PANX1 (red star) interactome consisting of plasma membrane (red), actin microfilament (blue) and microtubule (green) associated proteins.

Following these validation steps, the banding patterns of the biotinylated proteins captured by streptavidin beads from BirA*-PANX1, compared to its BirA* and Myc-PANX1 negative controls, were examined by western blotting. In both Rh18 and Rh30 cells, the BirA* biotin ligase activity was evident by the intense bands detected by fluorochrome conjugated streptavidin (Fig. 3A, B). Importantly, BirA*-PANX1 exhibited different banding patterns of biotinylated proteins compared to those biotinylated by BirA* alone (Fig. 3A, B). Rh18 and Rh30 cells were then treated with cumate to induce BirA*-PANX1 expression, or Myc-PANX1 as a background control, and exposed to biotin for 24 h to allow proximity biotinylation by BirA*. Cells were lysed and biotinylated proteins were captured using streptavidin-conjugated beads. The captured proteins were digested on-bead and immediately submitted for HPLC-ESI-MS/MS. A total of 240 and 238 proteins labeled by BirA*-PANX1 were identified from Rh18 and Rh30 cells, respectively (complete list of interactors identified by BioID is presented in Supplementary Table S1). Interactors were ranked by the unique peptide scores, which were calculated using Myc-PANX1 as the background control, indicating relative abundance of the peptides biotinylated by BirA*-PANX1. The top 50 interactors from Rh18 and Rh30 cells are shown in Fig. 3C, D, respectively. Most notably, 43 out of the top 50 candidate hits were shared between Rh18 and Rh30 cells, suggesting a high degree of confidence in the targets identified (Fig. 3E). STRING Functional Protein Association Network analysis and GO classification of the overlapped 43 protein hits presented a network of PANX1 interactors consisting of plasma membrane and cytoskeleton-associated proteins in RMS (Fig. 3F). Further analysis of the 14 mutually exclusive proteins (7 from each cell line (Fig. 3E)) showed that they are phosphoproteins with localizations in the cytosol, cytoskeleton, and plasma membrane. Taken together, these data suggest that PANX1 primarily interacts with proteins with functions localized in or proximal to the plasma membrane of both eRMS and aRMS cells.

### Online databases for searching the PANX1 transcriptome and interactome

To provide access to the information on the individual transcripts regulated in PANX1-expressing cells, as well as PANX1 interactors, we have generated user-friendly public online databases using RStudio equipped with the Shiny package. These databases can be freely accessed at http://bigbear.med.uottawa.ca:2000 and have been designed to toggle between the transcriptomic and proteomic data with a dropdown menu. The individual gene symbol and Ensemble gene ID or protein symbol in their respective databases can be freely searched. The gene hits are displayed with their Log_2_ fold change, *P* and *Q* values while the protein hits are displayed with their background-corrected scores (BirA*- or Myc-PANX1-expressing cells as negative controls), and these parameters can all be freely adjusted to meet individual specific search interests. When using our databases, kindly acknowledge the current manuscript.

### Proteomic analysis from Co-IP performed using PANX1-enriched membrane fractions

**Fig. 4:**
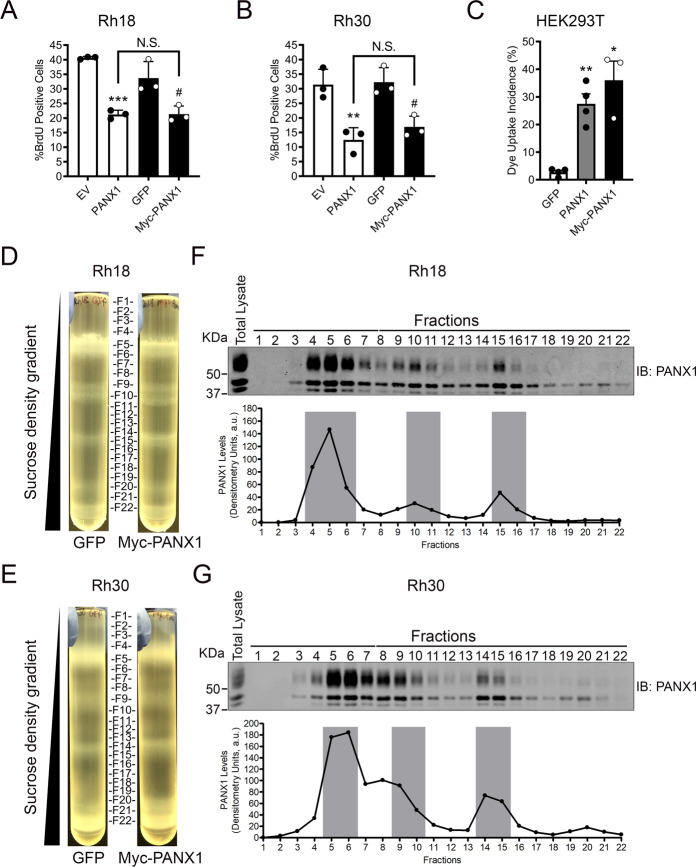
Enrichment of PANX1 by subcellular fractionation. BrdU incorporation of wild-type Rh18 (eRMS) (**A**) and Rh30 (aRMS) (**B**) cells expressing PANX1 or Myc-PANX1 and respective empty vector (EV) or GFP controls. Results are expressed as mean ± s.d. of three independent experiments. **P < 0.01 and ***P < 0.001 compared to EV, ^#^*P* < 0.05 compared to GFP. N.S. not significant. **C** Sulforhodamine B dye uptake induced by mechanical stimulation with HEK293T cells transiently expressing GFP, PANX1, or Myc-PANX1. Results are expressed as mean ± s.d. of four independent experiments. *P < 0.05 and **P < 0.01 compared to GFP. Stable Rh18 and Rh30 cells expressing Myc-PANX1 or its GFP control vector were homogenized and separated by a sucrose gradient by ultra-highspeed centrifugation to enrich Myc-PANX1-containing subcellular fractions. The Myc-PANX1-enriched fractions or their corresponding GFP control fractions were pooled and subjected to co-immunoprecipitation by anti-Myc antibodies. The co-IP’ed samples were further analyzed by HPLC-ESI-MS/MS. Representative images of Rh18 (**D**) and Rh30 (**E**) cell lysates separated by a sucrose density gradient. Subcellular fractions of Rh18 (**F**) and Rh30 (**G**) cell lysates containing Myc-PANX1 were analyzed by western blotting and the quantification of Myc-PANX1 levels are shown below. The Myc-PANX1 enriched fractions, indicated by the shaded areas, and their corresponding GFP control fractions were combined separately for co-IP by anti-Myc antibodies and the subsequent HPLC-ESI-MS/MS analysis. Results are from two independent experiments.

Fowler et al. have previously developed and optimized a method to effectively isolate and enrich subcellular compartments containing integral membrane proteins using a sucrose density gradient prior to co-IP followed by LC–MS/MS analysis [33, 34]. This complementary method was used here to isolate Myc-PANX1-enriched membrane microdomains from Rh18 and Rh30 cells prior to co-IP in an attempt to eliminate potential false positive interactors from BioID. Previously used in various contexts, the Myc tag showed no impact on PANX1 channel activity [17, 35], trafficking [36] and localization [37]. Accordingly, transient expression of PANX1 and Myc-PANX1 inhibited Rh18 (Fig. 4A) and Rh30 (Fig. 4B) cell proliferation to a similar extent, as compared to their respective negative controls. In addition, transient expression of both PANX1 and Myc-PANX1 in HEK293T cells induced comparable levels of dye uptake, which were significantly higher than that of the GFP control (Fig. 4C). Following these verification steps, whole lysates of stable Rh18 (Fig. 4D) and Rh30 (Fig. 4E) cells expressing Myc-PANX1 or GFP under the control of the cumate-inducible system were separated on a sucrose gradient from which a total of 22 matching fractions were collected. Fractions with high levels of Myc-PANX1 (indicated by shaded area; Fig. 4F, G) were pooled and used for co-IP followed by HPLC-ESI-MS/MS analysis.

**Fig. 5:**
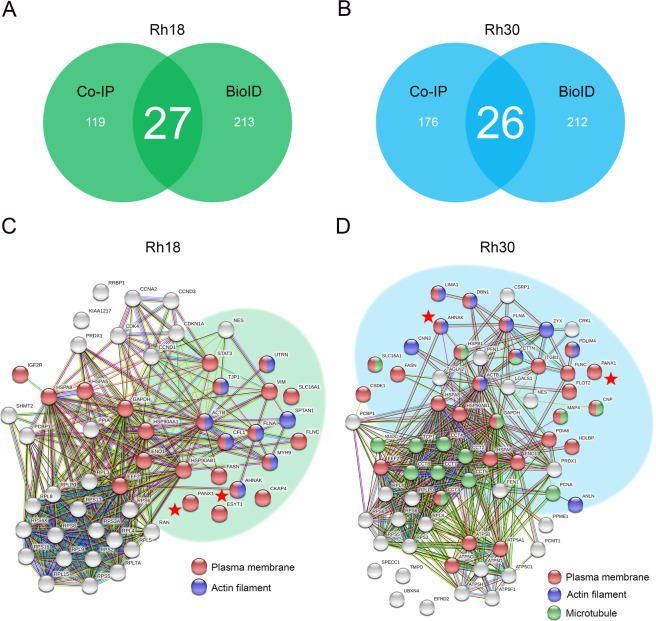
The overlapping hits identified from BioID and Co-IP using PANX1-enriched subcellular fractions revealed an interactome consisting of plasma membrane-, actin filament-, and microtubule-associated proteins. The total protein hits in Rh18 (eRMS) and Rh30 (aRMS) cells identified by both BioID and co-IP using Myc-PANX1 enriched fractions were analyzed. Venn diagrams show 27 and 26 overlapping protein hits between the two HPLC-ESI-MS/MS approaches with Rh18 (**A**) and Rh30 (**B**) cells, respectively. STRING protein interaction network analysis of the overlapping hits from Rh18 (**C**) and Rh30 (**D**) cells. The proteins in the network are labeled according to their respective cell component GO terms: plasma membrane (red), actin microfilament (blue) and microtubules (green). PANX1 and the top hit AHNAK are marked with a red star. The shaded areas show clusters of plasma membrane associated protein hits.

A total of 146 and 202 protein hits were identified by HPLC-ESI-MS/MS from Rh18 and Rh30 cells, respectively. These proteins were selected according to their scores, which were derived from the relative fold change of unique peptides from Myc-PANX1 samples to the GFP controls, and only proteins with scores above 1 were selected for the downstream analyses. The protein hits were cross-referenced with the PANX1 interactome identified from BioID and revealed 27 and 26 overlapped protein hits, where AHNAK, UTRN, and MYH9 were amongst the highest enriched hits, in both the BioID and co-IP approaches in Rh18 (Fig. 5A) and Rh30 (Fig. 5B) cells, respectively. These 27 and 26 PANX1 interactors in Rh18 (Fig. 5C) and Rh30 (Fig. 5D) cells are predicted in clusters of interacting proteins consisting of plasma membrane-, actin filaments- and microtubule-associated proteins.

### PANX1 tumor inhibitory function in RMS is dependent on its interaction with AHNAK

**Fig. 6:**
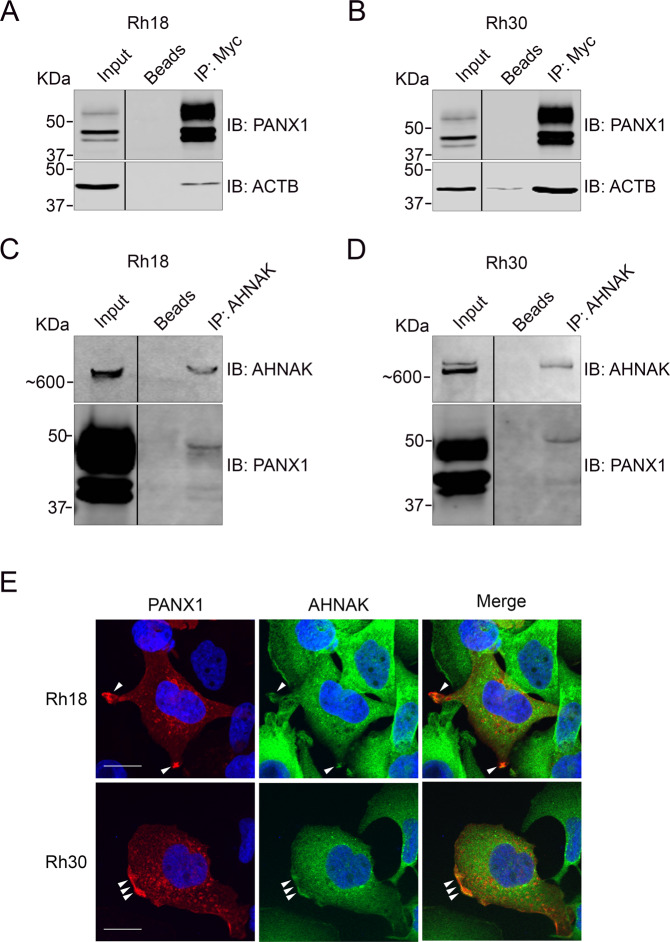
PANX1 interacts with ACTB and AHNAK. Stable Rh18 (eRMS) and Rh30 (aRMS) cells were induced to express PANX1 or Myc-PANX1 for 48 h and whole cell lysates were subjected to co-IP followed by western blotting analyses. The known PANX1 binding partner, ACTB, was pull-downed with Myc-PANX1 in Rh18 (**A**) and Rh30 (**B**) lysates using anti-Myc antibodies. In addition, PANX1 was pull-downed with AHNAK in both Rh18 (**C**) and Rh30 (**D**) lysates using anti-AHNAK antibodies. All western blots are representative of three independent experiments. **E** Immunofluorescence confocal laser microscopy showing colocalization (arrowheads) of transiently expressed PANX1 (red) and endogenous AHNAK (green) in Rh18 and Rh30 cells. DAPI-stained nuclei are shown in blue. Bar = 20 µm.

To start assessing the functional relevance of the PANX1 interactome discovered here, we first wanted to confirm the physical interaction of PANX1 with AHNAK; the highest ranked protein hit from BioID and detected by co-IP using enriched subcellular fractions. AHNAK is a large scaffold protein [24, 38, 39] that has been shown to regulate the proliferation, migration and invasion of mesothelioma [25], triple negative breast cancer [27] and pancreatic cancer cells [40]. However, the functional role of AHNAK in RMS and its interaction with PANX1 were unknown. Co-IP was performed using whole lysates of Rh18 and Rh30 cells expressing PANX1. ACTB was chosen as a positive control since PANX1 has been shown to interact with components of actin filaments [41]. ACTB was also identified as a PANX1 interactor from our BioID and subcellular fractionation/co-IP results. As expected, ACTB was co-immunoprecipitated with Myc-PANX1 in Rh18 (Fig. 6A) and Rh30 (Fig. 6B) cells. PANX1 was also pulled-down with endogenous AHNAK in both Rh18 (Fig. 6C) and Rh30 (Fig. 6D) cells and was not detected when lysates were incubated with beads alone; thereby further validating this novel PANX1 interactor.

PANX1 is localized both in intracellular compartments and at the plasma membrane in Rh18 and R30 cells [10]. As AHNAK has been reported in many subcellular localizations such as the nucleus [42, 43], cytoplasm [44–47], and plasma membrane [38, 44, 47–49] including pseudopodia [50], immunofluorescence confocal microscopy was performed to examine in which cellular compartment PANX1 and AHNAK may interact. Confocal microscopy images (Fig. 6E) of Rh18 and Rh30 transiently expressing PANX1 showed PANX1 and AHNAK mainly in intracellular compartments but also at, or associated with, the plasma membrane. Notably, a pool of PANX1 and AHNAK were found localized at the plasma membrane within structures resembling pseudopodia [50] (arrowheads), suggesting that a population of PANX1 and AHNAK interact in specialized plasma membrane compartments.

**Fig. 7:**
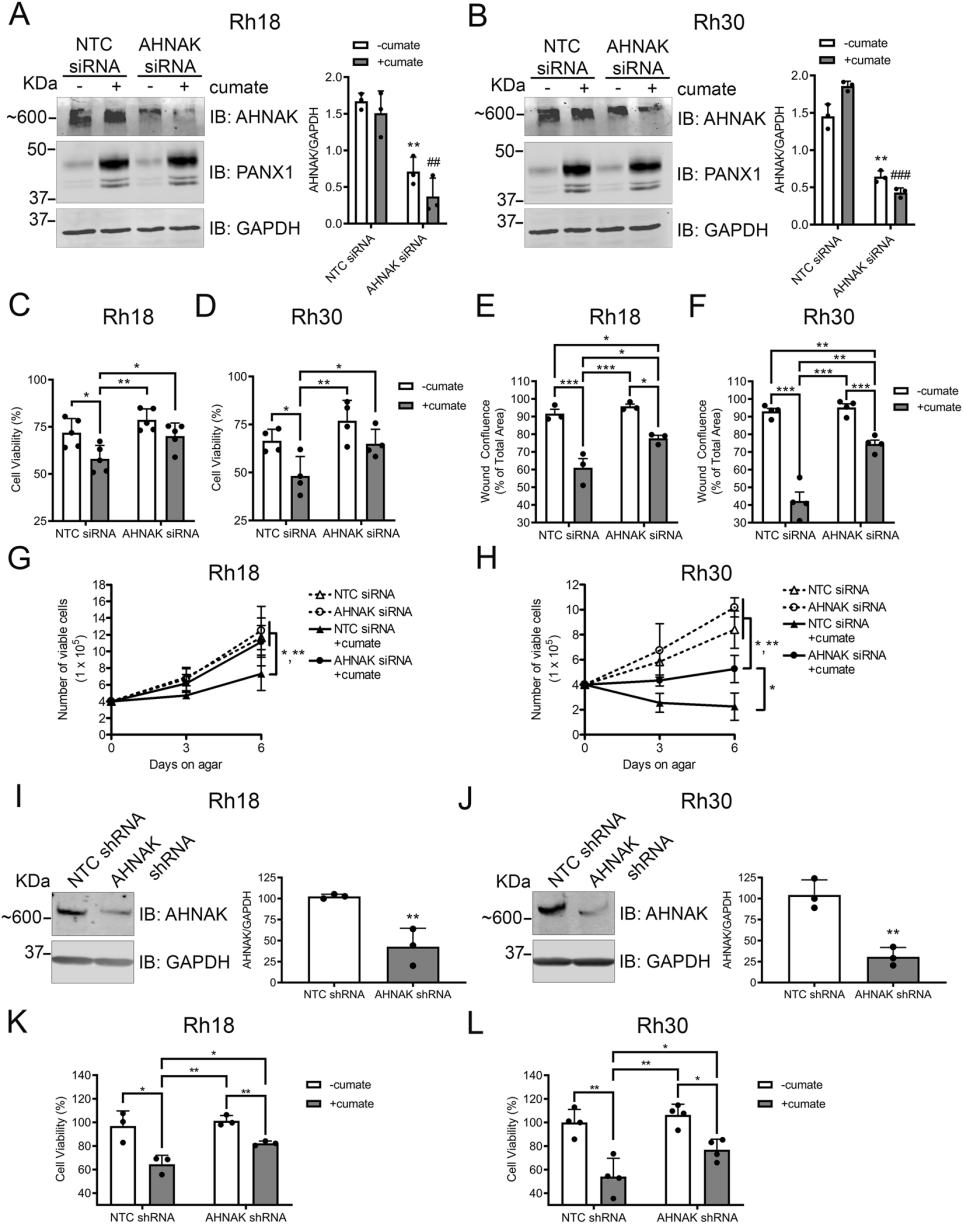
AHNAK knockdown significantly reversed the PANX1-induced suppression of malignant properties in RMS cells. Rh18 (eRMS) and Rh30 (aRMS) cells were transiently transfected with siRNA targeting AHNAK or a scrambled siRNA non-targeting control (NTC). Representative western blots of Rh18 (**A**) and Rh30 (**B**) cells and their respective quantifications (*n* = 3) of AHNAK levels 72 h following siRNA-mediated knockdown (KD). GAPDH was used as a loading control. **P < 0.01 compared to NTC siRNA (−cumate); ^##^*P* < 0.01 and ^###^*P* < 0.001 compared to NTC siRNA (+cumate). PANX1 expression was induced with 30 µg/mL of cumate 48 h post AHNAK KD and then subjected to Alamar blue viability, scratch wound migration, and soft agar anoikis assays. Alamar blue assay of Rh18 (*n* = 5) (**C**) and Rh30 (*n* = 4) (**D**) showing percent (%) cell viability normalized to NTC siRNA (-cumate) of the respective cell line. *P < 0.05 and **P < 0.01. Wound closure of Rh18 (*n* = 3) (**E**) and Rh30 (*n* = 4) (**F**) cells, were monitored for 60 or 80 h, respectively. The confluence of the wound area at the endpoint is shown as a percentage of the NTC siRNA (−cumate) from the respective cell line. *P < 0.05, **P < 0.01 and ***P < 0.001. Rh18 (*n* = 4) (**G**) and Rh30 (*n* = 3) (**H**) cells were grown in suspension for 6 days and the number of viable cells counted by Trypan Blue dye exclusion assay on days 0, 3, and 6 are shown. Day 0 denotes the time of cell seeding on soft agar. *P < 0.05 and **P < 0.01. Stable Rh18 and Rh30 cells were treated with 50 ng/µL doxycycline for 96 h to induce the expression of AHNAK shRNA or its NTC shRNA and then analyzed by western blotting to assess AHNAK KD efficiency. Representative western blots and their respective quantifications (*n* = 3) of AHNAK levels in Rh18 (**I**) and Rh30 (**J**). GAPDH was used as a loading control. **P < 0.01 compared to NTC shRNA. For Alamar blue viability assay, stable Rh18 and Rh30 cells were treated with 30 µg/mL of cumate for 48 h to allow PANX1 expression post shRNA-mediated AHNAK KD. Results are expressed as percent (%) cell viability of NTC shRNA of either Rh18 (*n* = 3) (**K**) or Rh30 (*n* = 4) (**L**) cells in the absence of PANX1 overexpression (−cumate). *P < 0.05 and **P < 0.01. All results are expressed as mean ± s.d.

To interrogate the functional role of this interaction, AHNAK was knocked down in PANX1-expressing Rh18 (Fig. 7A) and Rh30 (Fig. 7B) cells. As PANX1 reduces RMS cell proliferation and migration, and sensitizes RMS cells to anoikis [10], we used assays reflective of each phenotype. Indeed, Rh18 and Rh30 transfected with AHNAK siRNA or the scramble non-targeting control (NTC) were treated with or without cumate to induce PANX1 expression [10] prior to Alamar Blue viability, scratch wound migration, or soft agar anoikis assays. We found that AHNAK knockdown abrogated the PANX1-mediated reduction in Rh18 (Fig. 7C) and Rh30 (Fig. 7D) cell viability seen in their respective NTC siRNA counterparts. A partial reversal of the PANX1-mediated reduction in migration of PANX1-expressing Rh18 (Fig. 7E) and Rh30 (Fig. 7F) cells was observed when AHNAK expression was knocked down. Reduction of AHNAK expression completely reversed the PANX1-mediated sensitization to anoikis in Rh18 (Fig. 7G) cells, while a partial effect was observed in Rh30 (Fig. 7H) cells, compared to their respective controls. To further confirm our findings, we used shRNA as an alternative AHNAK knockdown strategy. Our cumate-inducible stable Rh18 (Fig. 7I) and Rh30 (Fig. 7J) cells were further engineered to express either NTC or AHNAK-targeting shRNA under a doxycycline switch [10, 51]. In accordance with our previous results, shRNA-mediated AHNAK knockdown in both Rh18 (Fig. 7K) and Rh30 (Fig. 7L) cells showed significant mitigation of PANX1-mediated reduction in cell viability. Altogether, these results indicate that the PANX1 tumor inhibitory function in RMS involves its interaction with AHNAK.

## Discussion

We had previously demonstrated that PANX1 alleviates RMS malignant properties [10]. However, the molecular mechanisms involved remained to be investigated. Here, we have taken a combination of unbiased genome-wide approaches to provide a comprehensive view of the PANX1 transcriptome and interactome in RMS. We found that PANX1 modulates the expression of genes involved in numerous BPs characteristic of cancer including, in descending order of significance, cell–cell adhesion, extracellular matrix disassembly, regulation of apoptosis, migration, and cellular morphology. Notably, regulation of apoptosis and cell migration were reflective of our previously reported PANX1-mediated inhibition of RMS migratory capacity and induction of apoptosis [10] and providing insights for potential responsible genes. For instance, the transcript of caspase 2, the most conserved caspase family member across species [52, 53], is found to be significantly upegulated in RMS cells expressing ectopic PANX1. Interestingly, PANX1 is a recognized target of caspase 3 and the cleavage of its c-terminal tail aids the progression of apoptosis [54]. We also found MAPK and Rap1 signaling pathways being significantly implicated in PANX1-expressing RMS cells. Induction of p38 MAPK signaling by a constitutively active mutant form of MAPK kinase 6 promotes terminal differentiation of RMS cells [55] and PKCα-mediated activation of MAPK cascades results in RMS cell growth arrest and differentiation [56]. Moreover, the crosstalk between MAPK and Rap1 signaling pathways has been shown to regulate metastasis of numerous cancers [57]. Future research may unveil whether PANX1 acts through MAPK and Rap1 signal transduction to reduce the proliferation and migration capacities of RMS cells [10]. To our surprise, the transcript and protein levels of Cx43 are increased by ectopic expression of PANX1 in RMS cells. As Cx43 overexpression in RMS has been shown to inhibit its proliferation and promote cellular fusion, an early step of myogenic differentiation [29], PANX1 may work in synergy with Cx43 to suppress RMS malignant phenotypes.

Notably, 43 of the top 50 protein hits from BioID between eRMS and aRMS cells are identical, suggesting that PANX1 inhibits the RMS malignant phenotype through similar mechanisms in both histological subtypes. The majority of the proteins in the PANX1 interactome are associated with the plasma membrane where PANX1 is known to localize and function [8]. However, due to the long half-life of PANX1 [11], plasma membrane bound proteins with weak or transient interactions with PANX1 may be overrepresented by extensive biotinylation. Moreover, as the number and spatial accessibility of lysine residues correlate with the level of biotinylation [31, 58], candidate proteins of large size may also be overrepresented. Nonetheless, these proteins may still be genuine interactors of PANX1 thus warranting further confirmation by Co-IP. The diverse localization pattern of the 14 mutually exclusive proteins in the cytosolic, cytoskeletal, and plasma membrane compartments suggest that these phosphoproteins were likely biotinylated during the trafficking of BirA*-PANX1 to the plasma membrane or were signaling molecules that traversed between multiple cellular compartments in RMS. The physical interaction between PANX1 and the actin cytoskeleton scaffold has been proposed to be responsible for the mechanosensitive opening of PANX1 channels in several cell types [59–62]. In addition, Panx1 has been suggested to regulate murine neurite migration and extension by remodeling their actin cytoskeleton through interaction with an actin cytoskeleton modulating protein, actin-related protein 3 (Arp3) [22]. We also found components of the actin cytoskeleton in our PANX1 interactome database, which together with our co-IP data of Myc-PANX1 and ACTB using whole-cell lysates, further confirmed this interaction in RMS. However, the ARP family of proteins was not found in our PANX1 interactome suggesting alternative mechanisms for PANX1 to regulate RMS cell migration for which the interaction of PANX1 with AHNAK may shed clues. Our data suggest that PANX1 interacts with AHNAK in specialized plasma membrane compartments resembling pseudopodia, which are known to be associated with tumor cell migration and invasion [27, 40, 50, 63, 64]. Interestingly, AHNAK has been implicated in the regulation of cell membrane cytoarchitecture, as well as pseudopod protrusion via interaction with Annexin A2, septin-9, and Ca^2+^-dependent S100 proteins [39, 50], which have both been shown to contribute to cancer metastasis [25–27, 50, 65]. Both Annexin A2 and the pseudopodia specific protein septin-9 [50] were found in the PANX1 interactome. Aside from migration, AHNAK has also been shown to regulate proliferation of several cancers [27, 63] and enhances the PKCα signaling pathway in fibroblast [66] which coincides with our RNA-seq data.

Collectively, we provide the first comprehensive transcriptomic and proteomic databases of PANX1 and further demonstrate their usefulness for exploring novel signaling mechanisms such as the newly described interaction of PANX1 with AHNAK. The functional dependence of AHNAK in PANX1-mediated regulation of RMS cell proliferation, migration, and anoikis provides the first insights into the molecular mechanism by which PANX1 inhibits RMS malignant properties. We expect that our transcriptomic and proteomic databases will foster new research and extend our current knowledge of the mechanism regulating PANX1, together with PANX1-mediated downstream signaling pathways in RMS and other cellular contexts.

## Material and methods

### Cells

Rh18 and Rh30 cell lines were from Dr. P. Houghton (St. Jude Children’s Hospital, Memphis, TN). HEK293T cell line was from the American Type Culture Collection. See “Supplementary Material and methods” for detailed information.

### Subcloning, transfection, and stable cell lines generation

pcDNA3.1-MCS-Bir*A(R118G)-HA was a gift from Kyle Roux (plasmid #36047, Addgene, Cambridge, MA) [31]. *PANX1* cDNA (Origene, Rockville, MD) was subcloned into pcDNA3.1-MCS-BirA*(R118G)-HA. PANX1-BirA*(R118G)-HA, and *Myc-PANX1* cDNA (Origene) were subcloned into the pCDH-CuO-MCS-EF1-GFP lentiviral vector (System Biosciences, CA). All constructs were verified by sequencing. See “Supplementary Material and methods” for detailed information.

Transfections were performed using Lipofectamine 2000 Reagent (Thermo Scientific, Waltham, MA). SparQ^TM^ Cumate Switch Inducible System (System Bioscience) was used to generate stable cell lines [10]. Cumate (System Bioscience) was used at 30 µg/mL.

### RT-qPCR

Stable Rh30 cells were treated and analyzed as described in “Supplementary Material and methods”.

### RNA sequencing and data analysis

Stable Rh30 cells were treated with cumate for 48 h. Total RNA was extracted using RNeasy Mini Kit (Qiagen, Germantown, MD) and submitted to Princess Margaret Genomics Centre (Toronto, ON, Canada) for RNA-seq analysis on an Illumina HiSeq2000 sequencing platform. See “Supplementary Material and methods” for detailed information.

### Western blotting

Cell lysates were obtained and analyzed as previously described [6, 67]. See “Supplementary Material and methods” for detailed information on antibodies and their dilutions.

### Dye uptake assay

Sulforhodamine B dye uptake assay was performed as previously described [10]. See “Supplementary Material and methods” for detailed information.

### 3D tumor spheroid assay

3D tumor spheroid assay was performed as previously described [10]. See “Supplementary Material and methods” for detailed information.

### BioID

BioID was performed according to Roux et al. [31]. See “Supplementary Material and methods” for detailed information.

### co-IP using enriched subcellular fractions

Subcellular fractionation from cells grown in five 15-cm dishes was performed according to Fowler et al. [33]. A total of 24 fractions were collected per sample. Myc-PANX1-enriched fractions were pooled and the buffer exchanged for IP lysis buffer (150 mM NaCl, 10 mM Tris-HCl, pH 7.4, 1 mM EDTA, 0.5% NP-40, and 1% Triton X-100) prior to performing the co-IP. See “Supplementary Material and methods” for detailed information.

### Mass spectrometry and proteomic analysis

Mass spectrometry was carried out at the Ottawa Institute of System Biology. See “Supplemental Material and methods” for detailed information.

### co-IP using whole-cell lysates

Cells were lysed using IP lysis buffer, as described above. Pre-cleared lysates were incubated with 5–8 µg of antibodies for 16 h, and then with 20 µL of freshly prepared protein A/G plus agarose beads (Thermo Fisher) for 60 min. Beads were washed, boiled in Laemmli buffer, and eluates submitted to western blotting.

### Confocal laser microscopy

Immunofluorescent labeling and acquisition of confocal images were described previously [10]. See “Supplementary Material and methods” for detailed information.

### Proliferation assay

Cells were subjected to a BrdU cell proliferation assay as previously described [10]. See “Supplementary Material and methods” for detailed information.

### RNA interference

Cells were transfected with 5 nM of Silencer^®^ Select siRNA targeting *AHNAK* (Sense: CCAUCUACUUUGACAACCUtt; Anti-sense: AGGUUGUCAAAGUAGAUGGtg) or its non-targeting control (NTC), Silencer^®^ Select Negative Control No. 1 (Life Technologies) for 72 h prior to analysis by western blotting for AHNAK levels or for 48 h prior to be subjected to various functional assays.

shRNA (TRCN0000130911) targeting *AHNAK* (Forward 5′–3′: ctagcGCACTTGAAGATGCCCAAGATCTCGAGATCTTGGGCATCTTCAAGTGCTTTTTTG; Reverse 5′–3′: gCGTGAACTTCTACGGGTTCTAGAGCTCTAGAACCCGTAGAAGTTCACGAAAAAACttaa) and or its NTC (Forward 5′–3′: ctagcGCGCGATAGCGCTAATAATTTCTCGAGAAATTATTAGCGCTATCGCGCTTTTTG; Reverse 5′–3′: gCGCGCTATCGCGATTATTAAAGAGCTCTTTAATAATCGCGATAGCGCGAAAAACttaa) were subcloned into EZ-Tet-pLKO-Hygro plasmid (a gift from Cindy Miranti, plasmid # 85972, Addgene), and packaged into lentiviruses using pMD2.G and psPAX2 packaging plasmids (gifts from Didier Trono, Addgene plasmids # 12259 and 12260) [51]. Cumate-inducible Rh18 and Rh30 cells [10] were used to generate the shRNA stable cells. Resulting cells were under cumate and doxycycline (50 ng/µL; Sigma-Aldridge) switch for PANX1 and shRNA expression, respectively.

### Alamar blue viability assay

Cells were incubated with 0.15 g/mL Alamar Blue (Sigma-Aldridge) for 2 h and read on a Synergy HTX plate reader (BioTek, VT) equipped with excitation;emission filter set at 530/25; 590/25 nm. See “Supplementary Material and methods” for detailed information.

### Migration assay

Migration assay was performed as previously described [10]. See “Supplementary Material and methods” for detailed information.

### Soft agar anoikis assay

Cells were seeded on 1% Noble agar (BD Biosciences, San Jose, CA) and viable cells counted [10]. See “Supplementary Material and methods” for detailed information.

### Data deposition

Raw fastq files from the RNA-seq used for bioinformatical analyses and the Shinny DE database can be accessed from Gene Expression Omnibus database (GSE144102). RAW spectra files from BioID and the Shinny DE database are deposited in the Center for Computational Mass Spectrometry (MassIVE MSV000084867).

### Statistics

Unpaired two-tailed Student’s *t* tests and analysis of variance followed by Tukey’s or Bonferroni *post hoc* tests were used. Results are given as mean ± s.d. Results with *P* < 0.05 were considered significant. Each experiment was performed at least three times (*n* = 3). The exact number of times each experiment was performed is indicated in the figure legends. The individual data points are also displayed on the graphs.

## Supplementary information

Supplemental Material and Methods

Supplemental Table S1

Supplementary Legends
