# Supplementary material for: Identification of pannexin 1-regulated genes, interactome, and pathways in rhabdomyosarcoma and its tumor inhibitory interaction with AHNAK
Source: Oncogene. 2021 Feb 9;40(10):1868–83. doi: 10.1038/s41388-020-01623-2 (PMC7946643; doi:10.1038/s41388-020-01623-2)
Supplement: Supplementary file 1 — Supplemental Material and Methods [file 41388_2020_1623_MOESM1_ESM.docx]

**SUPPLEMENTAL MATERIAL AND METHODS**

***Cell Lines and Culture Conditions***

Patient-derived rhabdomyosarcoma cell lines Rh18 (eRMS) and Rh30 (aRMS) lines, representing each of the main RMS subtype, were obtained from Dr. P. Houghton (St. Jude Children’s Hospital, Memphis, TN) whereas HEK293T cell line was purchased from the American Type Culture Collection. The parental Rh18 and Rh30 cell lines were cultured in RPMI-1640 media supplemented with 10% FBS and 1% penicillin/streptomycin. The doubling time of parental Rh18 and Rh30 cells are 34.0 *±* 1.5 and 38.8 *±* 5.6 hours, respectively. Stable inducible Rh18 and Rh30 cell lines for GFP, PANX1, Myc-PANX1 and BirA*-PANX1 expression were cultured in the same RPMI-1640 media with additional 100 µg/mL geneticin and 2 µg/mL puromycin. Hygromycin was further added at 50 µg/mL for maintaining stable Rh18 and Rh30 cells lines for AHNAK or NTC shRNA expression. HEK293T cell line was cultured in Dulbecco's modified Eagle's Medium (DMEM) media supplemented with 10% FBS and 1% penicillin/streptomycin. All cell cultures were incubated at 37°C, 5% CO_2_ and all tested negative for mycoplasma.

***Subcloning, Transfection and Generation of Stable Cell Lines***

BioID plasmid pcDNA3.1-MCS-BirA*(R118G)-HA was a gift from Kyle Roux (plasmid #36047, Addgene, Cambridge, MA)^1^. *PANX1* cDNA (Origene, Rockville, MD) was subcloned into pcDNA3.1-MCS-BirA*(R118G)-HA. *Myc-PANX1* cDNA (Origene, Rockville, MD) and *PANX1-BirA*(R118G)-HA (BirA*-PANX1)* were then subcloned into the pCDH-CuO-MCS-EF1-GFP lentiviral vector (System Biosciences, Palo Alto, CA). Stable cumate-inducible cell lines were generated using SparQ Cumate Switch Inducible System (System Bioscience) through lentiviral infection. Briefly, Rh18 and Rh30 cells stably expressing CymR repressor (pCDH-EF1-CymR-T2A-Neo lentivector) were transduced with pCDH-CuO-Myc-PANX1-EF1-GFP (Myc-PANX1) and pCDH-CuO-BirA*-PANX1-EF1-GFP (BirA*-PANX1) lentiviruses and selected using puromycin. Stable cumate-inducible cell lines for PANX1 expression or their empty plasmid control (GFP) were generated previously^2^. Expression of PANX1, Myc-PANX1, and BirA*-PANX1 was induced with 30 µg/mL cumate (System Bioscience).

Transient transfections were performed using Lipofectamine 2000 Reagent (Thermo Scientific, Waltham, MA).

***RNA Sequencing and Data Analysis***

Inducible stable Rh30 cells were treated with cumate for 48 hours. Total RNA was extracted using RNeasy Mini Kit (Qiagen, Germantown, MD) and submitted to Princess Margaret Genomics Centre (Toronto, ON, Canada) for RNA-seq analysis on an Illumina HiSeq2000 sequencing platform. The RNA Integrity Number (RIN) was 10 for all sequenced samples. Total purity filtered reads sequenced generated were 80,991,098, 93,328,541, and 156,294,622 for the biological replicates of GFP control samples, and 89,903,239, 102,149,600, and 105,348,940 for the matching replicates of PANX1-expressing samples. Overall read quality was checked using FASTQC v.0.11.2 RNA-SeQC (v1.1.7) software. Raw sequence data, in the form of FASTQ files, was aligned to the human genome (hg19, iGenome GTF definition file) using the BOWTIE/TOPHAT pipeline (BOWTIE v2.2.3, TOPHAT 2.0.13)^3^. Accessory programs for the alignment stage included SAMTOOLS (v1.0) and CUTADAPT (v1.7.1). Transcript assembly, abundance estimation, and tests for differential regulation were done using CUFFLINKS (v2.2.1). Genes with a false discovery rate (q value) < 0.05 were considered significantly changed, and genes with a log_2_ fold change > 1 or < -1 were denoted as up- or down-regulated. Gene Ontology (GO) enrichment and Kyoto Encyclopedia of Genes and Genomes (KEGG) pathway analyses were performed using the Database for Annotation, Visualization and Integrated Discovery (DAVID) v6.8 (https://david.ncifcrf.gov/) against a background species of the *H. sapiens*^4^. Enrichment of GO Biological Process (BP) terms and KEGG pathways were considered statistically significant when the Fisher’s exact p-values were < 0.05.

***RT-qPCR***

Inducible stable Rh30 (aRMS) cells were treated with cumate for 48 hours prior to extraction of total RNA using RNeasy Mini Kit (Qiagen). Total RNA was DNase-treated with TURBO DNA-free^TM^ kit and reverse transcribed into cDNA using High-Capacity cDNA Reverse Transcription Kit (Thermo Fisher). qPCR was performed with SsoAdvanced SYBR Green^TM^ Super Mix using 50 ng cDNA with the following gene-specific validated primers (Origene): *MMP2*: forward 5’- AGCGAGTGGATGCCGCCTTTAA-3’; reverse 5’- CATTCCAGGCATCTGCGATGAG-3’, *TRAF2*: forward 5’- GAGCAGAAGGTCTTGGAGATGG-3’; reverse 5’- GCAGACACATCTTGTAGCCGTAC-3’, *APOBEC2*: forward 5’- AGAACCTGGACGACCCTGAGAA-3’; reverse 5’- CAACCACATAGCAGAGGAAGGTC-3’, *MARCKS*: forward 5’- CTCCTCGACTTCTTCGCCCAAG-3’; reverse 5’- TCTTGAAGGAGAAGCCGCTCAG-3’, *GAPDH*: forward 5’- GTCTCCTCTGACTTCAACAGCG-3’; reverse 5’- ACCACCCTGTTGCTGTAGCCAA-3’. Relative expression was determined using the comparative Ct method.

***Western Blotting***

Lysates were separated by SDS-PAGE, transferred to PVDF membranes, and probed with anti-Cx43 (1:2000; Sigma-Aldrich, Cat#: C6219), PANX1 (Sigma-Aldrich, Cat#: HPA016930), Myc (Cell Signaling Technologies, Danvers, MA, Cat#: 2276), ACTB (Santa Cruz, TX, Cat#: sc-47778), AHNAK (AVIVA Systems, San Diego, CA, Cat#: OAEE00010), or GAPDH (1:5000; Advanced ImmunoChemical, Long Beach, CA, Cat#: 2RGM2). Alexa 680- (Thermo Scientific, Cat#: A-31553) or infrared fluorescent-labeled secondary antibodies IRDye 800 (Rockland Immunochemicals, Pottstown, PA, Cat#: 610-132-121) were used. For detection of biotinylated proteins, Streptavidin-Alexa Fluor 790 (Jackson ImmunoResearch, PA, Cat#: 016-650-084) was utilized. Immunoblots were processed and quantified using the Odyssey infrared-imaging system (LI-COR Biosciences, Lincoln, NE). All primary antibodies were used at 1:1000 unless otherwise specified. All secondary antibodies were used at 1:5000.

***Dye Uptake Assay***

HEK293T cells were transiently transfected with GFP, PANX1, BirA*-PANX1, or Myc-PANX1 constructs in 35 mm collagen-coated dishes. At 48 hours post-transfection, cells were washed twice with sterile Dulbecco’s-PBS (PBS plus 0.1 g/l CaCl_2_ anhydrous and 0.1 g/l MgCl_2_.6 H_2_O, pH 7.4) on ice and mechanically stimulated with a continuous drip of 800 µl of sulforhodamine B dye (2 mg/ml prepared in Dulbecco’s-PBS) released from a height of 2.5 cm above the dish. The stimulation was repeated two times and cells were then incubated with the dye on ice for 5 minutes. The cells were washed 10 times with cold Dulbecco’s-PBS prior to examining the dye uptake under a fluorescent microscope (Olympus IX51). Four fluorescent and their corresponding phase-contrast image fields were collected at the drip target site using a 20x objective. The % of dye uptake incidence was calculated as (the number of cells that took the dye/the total number of cells) x 100 for each of the four field and then averaged for each experiment (n=4)^2^.

***3D Tumor Spheroid Assay***

3D spheroid assay was performed and quantified using IncuCyte ZOOM Live Cell Imaging System (Essen Bioscience) and its accompanying software as previously described^2^. Briefly, stable Rh18 and Rh30 cells expressing PANX1, BirA*-PANX1 or empty plasmid (GFP) were pre-treated with cumate for 16 hours and then seeded in ultralow adhesion (ULA) 96-well plates at 5,000 cells per well. Mean image fluorescence, a surrogate measurement of 3D tumor growth, was quantified using IncuCyte ZOOM Live Cell Imaging System every 2 hours for 200 hours.

***BioID***

BioID was performed according to Roux *et al.*^5^. Briefly, stable Rh18 (eRMS) and Rh30 (aRMS) were treated with 30 µg/mL cumate for 24 hours to induce expression of BirA*, Myc-PANX1 and BirA*-PANX1. The cells were then further incubated with 50 µM biotin for another 24 hours before harvesting, as BirA* typically takes 16-18 hours to reach saturation in these conditions^1^ and Panx1 has a half-life of about 21 hours^9^. The cells were sonicated using a VCX 130 Sonic Vibra-Cell^TM^ sonicator (Sonics & Materials, TC) set to 20% amplitude and 5 s/pulse till the lysate became clear. The biotinylated proteins were captured by incubating with Pierce™ NeutrAvidin™ Agarose (Thermo Scientific) beads for 18 hours at 4 ℃ on a rotator. To prepare for mass spectrometry, the beads were washed and re-suspended in 200 µL of 50 mM ammonium bicarbonate, incubated in 10 mM TCEP at 40 ℃ for 30 minutes and then reduced in 20 mM iodoacetamide for 30 minutes in the dark at 25 ℃. On-bead digestion was performed by adding 1/20 total sample volume of MS grade trypsin protease (Thermo Scientific) and incubating for 16 hours at 37 ℃ on a rotator. The reaction was stopped by adding 2% v/v formic acid and the samples were immediately submitted for LC-MS/MS analysis.

***co-IP using Enriched Subcellular Fractions***

Subcellular fractionation was performed according to Fowler *et al.*^6^ with modifications to accommodate cells as starting analytes. Stable Rh18 (eRMS) and Rh30 (aRMS) cells were grown to 90% confluence in 15 cm dishes and then treated with 30 µg/mL cumate for 48 hours to induce Myc-PANX1 expression. Their respective GFP-expressing cells were grown and treated alongside as controls. The cells were collected using a cell scraper and each sample was pooled from five 15 cm dishes. The cells were transferred into a Dounce homogenizer (Sigma-Aldrich, MO) and 100 strokes were applied to complete the lysis. The nuclei and cellular debris were removed by centrifuging at 800x g for 5 minutes at 4 ℃. The post nuclear supernatants (PNS) were then concentrated down to 1.4 mL using Amicon Ultra-15 Centrifugal Filter Units with a 10 kDa molecular weight cut-off (Millipore, MA) by centrifuging at 4000x g for 90 minutes at 4 ℃. The concentrated PNS were mixed with OptiPrep™ Density Gradient Medium (OP) (Sigma-Aldrich) to reach a final OP concentration of 30%. The 30% OP/PNS solution was added into polyallomer centrifuge tubes (VWR, PA) on which a discontinuous gradient of 25%, 20%, 17.5% and 10% of OP were overlaid sequentially. The gradients were assembled into a SW41 Ti rotor (Beckman Coulter, IN) and centrifuged at 200,000x g for 16 hours at 4 ℃ (acceleration = 1, deceleration = 0). A total of 22 fractions were collected from each sample and the Myc-PANX1 enriched fractions and their corresponding fractions in the GFP controls were pooled according the Western blotting results. The buffer of the pooled lysates was exchanged for IP lysis buffer (150 mM NaCl, 10 mM Tris-HCl, pH 7.4, 1 mM EDTA, 0.5% NP-40, and 1% Triton X-100) by three concentration and re-suspension cycles using the centrifugal devices and 2.5 mg of each pooled lysates were subject to co-IP. Each lysate was pre-cleared with 50 µL of beads, then added into 100 µL of agarose beads pre-adsorbed with 5 ug of anti-Myc antibodies and incubated on a rotator for 16 hours at 4 ℃. The beads were prepared for LC-MS/MS analysis as described above.

***Mass Spectrometry and Proteomic Analysis***

Mass spectrometry was carried out at the OISB (Ottawa Institute of System Biology). Briefly, the trypsin-digested peptides were desalted on Sep-Pak^®^ C18 columns (Waters, MA), dried down in SpeedVac (Thermo Scientific) and reconstituted in 0.5% (v/v) formic acid before analysis by high-performance liquid chromatography/electrospray ionization tandem mass spectrometry (HPLC-ESI-MS/MS). The acquired MS/MS spectra were searched against the human International Protein Index (IPI) protein sequence database (v3.85) using Maxquant with the label free quantitation (LFQ) option and a biotin variable modification was added for BioID^7^. The false discovery rate (FDR) was set to ≤ 1% on both protein and peptide level. Quantification was performed using normalized LFQ intensity. The number of unique peptides of each protein was further scored and ranked using the respective background controls; for BioID, the unique peptide number from BirA*-PANX1 was divided by those from Myc-PANX1 + 0.1 for each protein and ranked according to this ratio, and for subcellular fractionation combined with co-IP, the unique peptide number from Myc-PANX1 was divided by those from GPF + 0.1 for each matched protein. A cut-off unique peptide score of 1 was set for proteins found by either BioID or subcellular fractionation combined with co-IP. Overlapped protein hits were identified using Venny v2.1.0 (<https://bioinfogp.cnb.csic.es/tools/venny/>). Protein-protein interaction networks of the overlapped hits were predicted by STRING v11.0 (<https://string-db.org/>) with the interaction score set to medium, the maximum interactors to be displayed in the 1^st^ shell set to 10, and the network clustering set to ‘no clustering’. GO (Cellular Component) terms plasma membrane (GO: 0005886), actin cytoskeleton (GO: 0015629) and microtubule cytoskeleton (GO: 0015630) were selected to highlight the interactors accordingly.

***Confocal Laser Microscopy***

Rh18 (eRMS) and Rh30 (aRMS) on glass coverslips were transfected with Myc-PANX1 for 48 hours prior to fixation in 3.7% PFA for 20 minutes at room temperature. The fixed cells were blocked and permeabilized in 2% BSA and 0.1% Triton X-100 for 45 minutes and then sequentially labeled with anti-PANX1 (1:200; Sigma-Aldrich, Cat#: HPA016930), Alexa Fluor 594 conjugated anti-rabbit IgG (1:500; Thermo Fisher, Cat#: A-11012), anti-AHNAK (1:50; AVIVA Systems, Cat#: OAEE00010), and Alexa Fluor 488 conjugated anti-mouse IgG (1:500; Thermo Fisher, Cat#: A-11001) for 45 minutes each with PBS washes in between. The cells were then mounted with DAPI-Fluoromount G^TM^ (Thermo Fisher) and viewed with an Olympus Fluoview FV-1000 Laser Confocal Microscope equipped with a 60X oil objective. Image acquisition was performed with the Fluoview software. All settings were kept constant during image acquisition.

***Proliferation Assay***

Rh18 (eRMS) and Rh30 (aRMS) cells were transfected with either PANX1 or Myc-PANX1 and their respective empty vector (EV) or GFP controls. Twenty four hours post-transfection, cells were incubated with 10 µM BrdU (Sigma-Aldrich) for either 3 hours (Rh18) or 1 hour (Rh30) and processed for immunohistochemistry^2^. Briefly, the cells were fixed on glass coverslips for 20 minutes at room temperature in 3.7% PFA, washed with PBS. The fixed cells were blocked with 2% BSA with 0.1% Trinton X-100 for 45 minutes, and then incubated in 2N HCl for 20 minutes to nature the nuclear DNA. The cells were labeled sequentially with anti-BrdU (1:200; Thermo Fisher, Cat#: B35128), Alexa Fluor 594 conjugated anti-mouse IgG (1:500, Thermo Fisher, Cat#: A-11032), anti-PANX1 (1:200; Sigma-Aldrich, Cat#: HPA016930) and Alexa Fluor 488 conjugated anti-rabbit IgG (1:500, Thermo Fisher, Cat#: A-11008) with PBS washes in between. The labeled cells were then mounted using DAPI-Fluoromount-G^TM^ (Thermo Fisher). Ten random field images per sample were taken using a fluorescent microscope equipped with a 20X objective and the number of BrdU positive nuclei from total transfected cells (positive for GFP- or Myc-staining) were counted and quantified.

***Alamar Blue Viability Assay***

For siRNA-based knockdown, stable Rh18 (eRMS) and Rh30 (aRMS) cells were seeded in 96 well plates at 10,000 cells per well. The cells were transfected with AHNAK siRNA or NTC for 24 hours and then treated with cumate for another 48 hours before analysis. For shRNA-based knockdown, the shRNA-stable Rh18 and Rh30 cells were incubated in 50 ng/mL doxycycline (Sigma-Aldridge) for 72 hours, then seeded in 96 well plates at 20,000 cells per well. The cells were treated with and without cumate for 48 hours before analysis. Doxycycline and cumate were refreshed daily. In both knockdown approaches, the cells were incubated in culture media with 0.15 g/mL Alamar Blue (Sigma-Aldridge) for 2 hours, then the plates were read on a Synergy HTX plate reader (BioTek, VT) equipped with excitation;emission filter set at 530/25; 590/25 nm.

***Migration Assay***

Stable Rh18 and Rh30 cell lines were transfected with AHNAK siRNA or the NTC in the presence of cumate of its vehicle control (H_2_O) for 48 hours before a uniform scratch was made using the 96-well WoundMaker device (Essen Bioscience). The cells were monitored using the IncuCyte ZOOM Live Cell Imaging System (Essen Bioscience) and the green fluorescence from constitutively expressed GFP was used to measure the % of wound confluence over the period of 60 (Rh18) or 80 (Rh30) hours by its accompanying software.

***Soft Agar Anoikis Assay***

Stable Rh18 and Rh30 cell lines were transfected with AHNAK siRNA or the NTC for 24 hours and then seeded on top of 1% Noble agar (BD Biosciences, San Jose, CA) at 400,000 cells/well with and without cumate^8^. The cells were counted on Days 0 (day of cell seeding on agar), 3 and 6 by Trypan Blue (Thermo Scientific) dye exclusion using the Countess^TM^ Automated Cell Counter (Life Technologies) following the manufacturer’s instructions.

**REFERENCES**

1 Roux KJ, Kim DI, Raida M, Burke B. A promiscuous biotin ligase fusion protein identifies proximal and interacting proteins in mammalian cells. *J Cell Biol* 2012; **196**: 801–10.

2 Xiang X, Langlois S, St-Pierre ME, Barré JF, Grynspan D, Purgina B *et al.* Pannexin 1 inhibits rhabdomyosarcoma progression through a mechanism independent of its canonical channel function. *Oncogenesis* 2018; **7**. doi:10.1038/s41389-018-0100-4.

3 Trapnell C, Roberts A, Goff L, Pertea G, Kim D, Kelley DR *et al.* Differential gene and transcript expression analysis of RNA-seq experiments with TopHat and Cufflinks. *Nat Protoc* 2012; **7**: 562–578.

4 Huang DW, Sherman BT, Lempicki RA. Systematic and integrative analysis of large gene lists using DAVID bioinformatics resources. *Nat Protoc* 2009; **4**: 44–57.

5 Roux KJ. Marked by association: Techniques for proximity-dependent labeling of proteins in eukaryotic cells. *Cell Mol Life Sci* 2013; **70**: 3657–3664.

6 Fowler SL, Akins M, Zhou H, Figeys D, Bennett SAL. The liver connexin32 interactome is a novel plasma membrane-mitochondrial signaling nexus. *J Proteome Res* 2013; **12**: 2597–2610.

7 Cox J, Mann M. MaxQuant enables high peptide identification rates, individualized p.p.b.-range mass accuracies and proteome-wide protein quantification. *Nat Biotechnol* 2008; **26**: 1367–1372.

8 Wolf SJ, Huynh T, Bryce NS, Hambley TW, Wakelin LPG, Stewart BW *et al.* Intracellular trafficking as a determinant of AS-DACA cytotoxicity in rhabdomyosarcoma cells. *BMC Cell Biol* 2011; **12**: 36.

9 Boassa, D, Qiu, F., Dahl G, Sosinsky G. Trafficking dynamics of glycosylated pannexin 1 proteins. Cell Commun Adhes 2008, **15**:119-132.
