# Supplementary material for: Identification of pannexin 1-regulated genes, interactome, and pathways in rhabdomyosarcoma and its tumor inhibitory interaction with AHNAK
Source: Oncogene. 2021 Feb 9;40(10):1868–83. doi: 10.1038/s41388-020-01623-2 (PMC7946643; doi:10.1038/s41388-020-01623-2)
Supplement: Supplementary file 3 — Supplementary Legends [file 41388_2020_1623_MOESM3_ESM.docx]

**Supplemental Table S1**

Complete list of all proteins identified by BioID. Proteins sharing identical peptides were placed in the same group in which the most abundant protein is presented with its protein and associated gene name. The unique peptides were only associated with their specific protein groups, which were used for downstream analysis.

**Supplemental Material and Methods**

Details on the material and various methods used in these studies can be found in the Supplemental Material and Methods.
